# Accessory gallbladder: a new anatomical variation arising from both left and right hepatic ducts

**DOI:** 10.1308/003588412X13373405385052

**Published:** 2012-09

**Authors:** S Hassan, AL Young, M Farooq, D Pai, M Gough

**Keywords:** Accessory gallbladder

## Abstract

We present a case of accessory gallbladder demonstrating an anatomical variation not previously reported. While rare, accessory gallbladders are an important consideration if a cholecystectomy is to be performed. We also present a brief literature review of accessory gallbladders.

Symptomatic duplicate gallbladders are very rarely encountered in clinical practice. Autopsy studies in humans have reported this congenital biliary anomaly as occurring in 0.03% of cases.^1^ We report a case of accessory gallbladder that demonstrates a new anatomical variation and review the available literature.

## Case history

An 83-year-old Punjabi woman presented to the emergency department with severe abdominal pain. She gave a one-month history of intermittent generalised abdominal pain that had become very severe in the day prior to admission, radiating to both shoulders. The pain was exacerbated by eating and was associated with nausea and vomiting. Her past medical history included subarachnoid haemorrhage, type 2 diabetes mellitus, hypertension and myocardial infarction.

**Figure 1:**
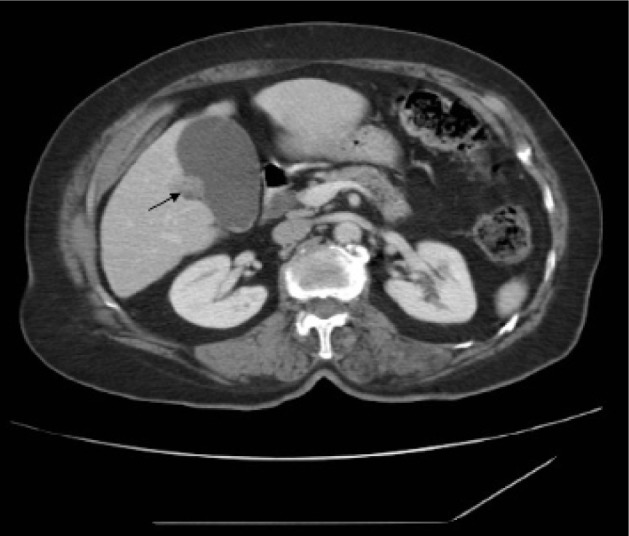
Computed tomography showing small focal lesion (arrow) originally suspected to be a neoplastic lesion

**Figure 2:**
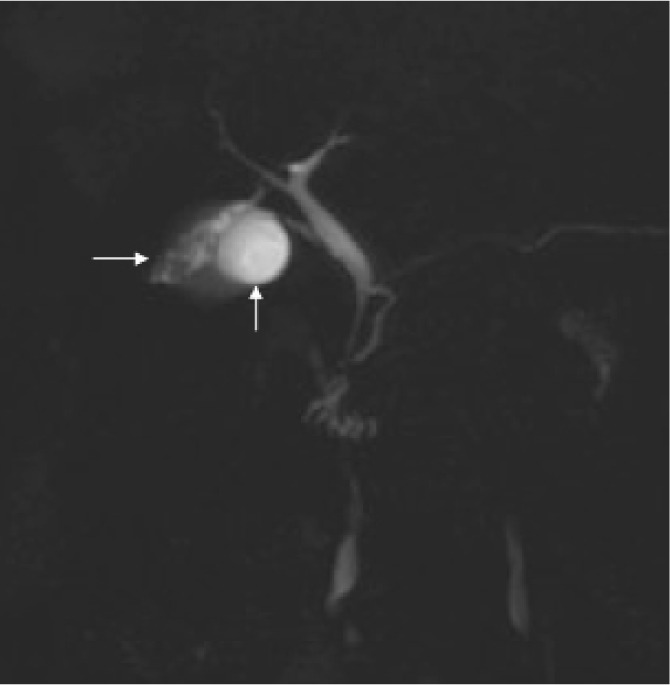
Maximum intensity projection image showing the two gallbladder structures (arrows)

**Figure 3:**
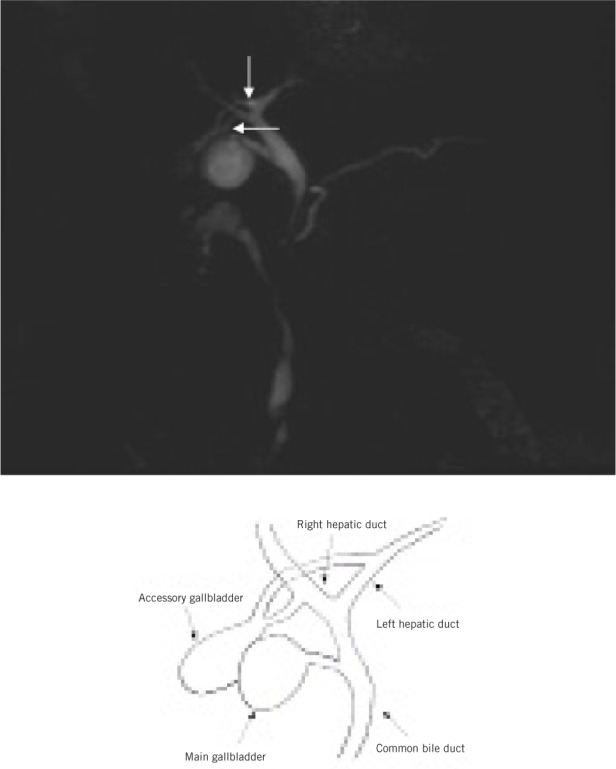
Maximum intensity projection image showing accessory gallbladder arising from both left and right hepatic ducts via two cystic ducts (arrows) with the accompanying diagram to illustrate the anatomy

Examination of her gastrointestinal system showed a soft abdomen but generalised tenderness most markedly over the epigastric region. All routine blood tests were within normal limits. Ultrasonography showed a small hyperreflective lesion on the gallbladder wall with no evidence of choledocholithiasis or cholelithiasis. Subsequently, computed tomography (CT) of the abdomen raised the possibility of a small focal neoplastic lesion in the gallbladder (Fig 1). Magnetic resonance cholangiopancreatography (MRCP) demonstrated an accessory gallbladder containing small calculi and connecting with both the left and right hepatic ducts via two small cystic ducts (Figs 2 and 3). The main gallbladder appeared normal and had its own cystic duct, which joined the common hepatic duct. A decision was made not to operate given the patient’s multiple medical co-morbidities and lack of persisting symptoms.

## Discussion

Duplication of the gallbladder is the result of a rare bifid embryonic diverticulum of the hepatic duct occurring during the fifth or early in the sixth embryonic week.^1^ Several case reports have been published over the years illustrating different types of double gallbladders and some authors have attempted to classify the multiple anatomical variations seen with these anomalies. The most commonly encountered were the Boyden,^1^ Gross^2^ and Harlaftis classifications.^3^

**Figure 4:**
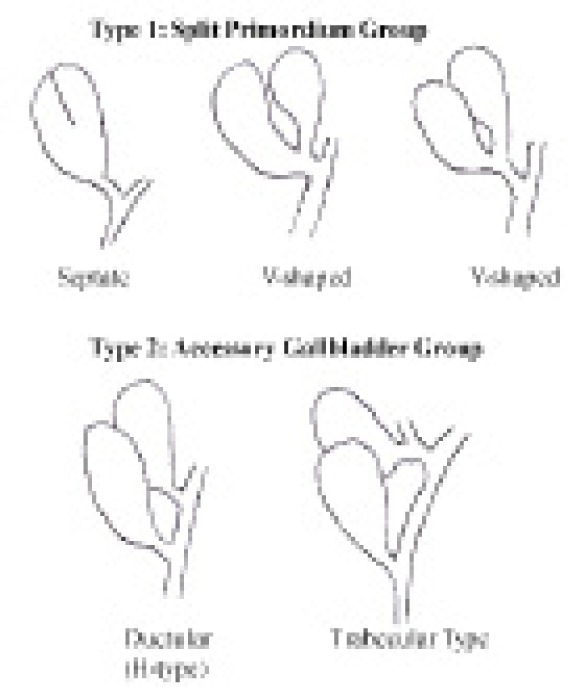
Harlaftis classification for accessory gallbladders (adapted from Kawanishi *et al*)^4^

Harlaftis *et al* classified double gallbladders into three categories. Type I was the split primordium group (septate, V-shaped and Y-shaped), type II was the accessory gallbladder group (H-type or trabecular pattern) and type III included any anomaly that could not be classified into the above two groups (Fig 4). According to this classification, the case described would represent a type 3 double gallbladder. Reports have been published of accessory gallbladders arising from either the left or right hepatic duct but to our knowledge none have shown an accessory gallbladder branching from both the left and right hepatic ducts.

Accurate visualisation of biliary anatomy is important to prevent iatrogenic injury to vascular and biliary structures. MRCP has the advantage of being a non-invasive tool and studies have suggested that the image resolution obtained during MRCP can be as accurate as its invasive counterparts.^5^ In relation to the above case, MRCP was able to rule out a neoplastic process that was suspected initially on CT. In cases where pre-operative MRCP has not been carried out, if an anomaly is encountered at laparoscopic cholecystectomy, then intra-operative cholangiography is mandatory to confirm the anatomy and reduce the risk of biliary injury.

## Conclusions

Although aberrant anatomy of the biliary tree is commonly found in clinical practice, encountering accessory gallbladders is very rare. This paper reports an anatomical variation not previously described and illustrates the increasing value of MRCP in evaluating gallbladder lesions accurately as well as giving important visualisation of the biliary tree prior to a laparoscopic cholecystectomy.
